# Zinc Iodide Dimethyl Sulfoxide Reduces Collagen Deposition by Increased Matrix Metalloproteinase-2 Expression and Activity in Lung Fibroblasts

**DOI:** 10.3390/biomedicines12061257

**Published:** 2024-06-05

**Authors:** Michael Roth, Bo Han, Chong Teck S’ng, Ba Xuan Hoang, Christopher Lambers

**Affiliations:** 1 University Hospital of Basel, University of Basel, 4031 Basel, Switzerland; 2 Cordoba-Nimni Tissue Engineering and Drug Discovery Lab, Department of Surgery, University of Southern California, Los Angeles, CA 90089, USA; 3 PersCellMed S’ng, 4450 Sissach, Switzerland; 4 Department of Pneumology, Ordensklinikum Linz Elisabethinen, Fadingerstr. 1, 4020 Linz, Austria; clambers@gmx.de

**Keywords:** extracellular matrix remodeling, gelatinases, collagen type I, zinc iodide dimethyl sulfoxide therapy, proliferation, chronic inflammatory lung diseases

## Abstract

Chronic inflammatory lung diseases are characterized by disease-specific extracellular matrix accumulation resulting from an imbalance of matrix metalloproteinases (MMPs) and their inhibitors. Zinc is essential for the function of MMPs, and zinc deficiency has been associated with enhanced tissue remodeling. This study assessed if zinc iodide (ZnI) supplementation through dimethyl sulfoxide (DMSO) modifies the action of MMPs in isolated human lung fibroblasts. The expression and activity of two gelatinases, MMP-2 and MMP-9, were determined by gelatin zymography and enzyme-linked immuno-sorbent assay (ELISA). Collagen degradation was determined by cell-based ELISAs. Collagen type I and fibronectin deposition was stimulated by human recombinant tumor growth factor β1 (TGF-β1). Untreated fibroblasts secreted MMP-2 but only minute amounts of MMP-9. TGF-β1 (5 ng/mL) reduced MMP-2 secretion, but stimulated collagen type I and fibronectin deposition. All the effects of TGF-β1 were significantly reduced in cells treated with ZnI-DMSO over 24 h, while ZnI and DMSO alone had a lower reducing effect. ZnI-DMSO alone did not increase MMP secretion but enhanced the ratio of active to inactive of MMP-2. ZnI alone had a lower enhancing effect than ZnI-DMSO on MMP activity. Furthermore, MMP-2 activity was increased by ZnI-DMSO and ZnI in the absence of cells. Soluble collagen type I increased in the medium of ZnI-DMSO- and ZnI-treated cells. Blocking MMP activity counteracted all the effects of ZnI-DMSO. Conclusion: The data suggest that the combination of ZnI with DMSO reduces fibrotic processes by increasing the degradation of collagen type I by up-regulating the activity of gelatinases. Thus, the combination of ZnI with DMSO might be considered for treatment of fibrotic disorders of the lung. DMSO supported the beneficial effects of ZnI.

## 1. Introduction

Tissue remodeling, especially the accumulation of collagen type I, is a characteristic of several chronic inflammatory diseases of the lung, such as asthma [1,2], chronic obstructive pulmonary disease (COPD) [1,3], and of lung fibrosis [4,5]. In all conditions, the predominantly up-regulated components are the tissue content of collagen type I and fibronectin. Why this pathology occurs in a disease-specific pattern in different lung compartments remains unknown [6].

For several years there has been discussion that the accumulation of extracellular matrix (ECM) in fibrotic aspects of chronic inflammatory lung diseases results from an imbalance between ECM building and degradation [7]. In lung fibroblasts, wood-fire smoke stimulated fibrotic events, including synthesis of transforming/tumor growth factor β1 (TGF-β1), collagens, fibronectin, and tissue inhibitors of matrix metalloproteinases (TIMPs), but reduced the expression of MMPs [8]. Interestingly, overexpression of MMP-2 in a mouse model reduced the development and progression of idiopathic pulmonary fibrosis, suggesting reducing ECM by means of gelatinases as a novel therapeutic concept [9]. On the other hand, the inhibition of MMP-9 was suggested as a new therapeutic target in idiopathic pulmonary fibrosis (IPF) [10].

Zinc is essential to the enzymatic activity of MMPs [11], and thus it is not surprising that zinc deficiency has been linked to fibrotic events in different organs including the lungs [12,13,14,15]. However, regarding fibrotic diseases of the lung, studies on the role of zinc deficiency or supplementation have been focused on its effect with regard to infection in cystic fibrosis [15,16,17]. Under experimental conditions, zinc deficiency increased inflammation and tissue remodeling in the lung [18].

However, the role of intracellular zinc on ECM metabolism via MMP-2 and MMP-9 in chronic inflammatory lung diseases has not been much investigated. We reported earlier that treatment of human airway smooth muscle cells with zinc salicylate-methylsulfonylmethane significantly reduced platelet-derived growth factor-BB (PDGF-BB)-induced collagen type I but not fibronectin deposition [19]. The compound also limited cell proliferation through activation of p21^(Waf1/Cip1)^ and the inhibition of ERK1/2 mitogen activated protein kinase as well as mTOR signaling [19].

Therefore, this study investigated if ZnI-DMSO supplementation modifies the expression and/or activity of two gelatinases, MMP-2 and MMP-9. In addition, the study assessed if ZnI-DMSO supplementation reduced the content of pro-inflammatory collagen type I in TGF-β1-stimulated human lung fibroblasts and whether this beneficial effect is mediated via MMPs.

## 2. Materials and Methods

### 2.1. Cells

Non-diseased primary human lung fibroblast cells were obtained from Lonza (cat#CC-2512, Lonza, Basel, Switzerland). Cells were expanded in RPMI-1640 supplemented with 10% fetal calf serum, 8 mM L-glutamine, 20 mM HEPES, and 1x on-essential amino acid mix, all from Gibco/BRL (Life Technologies Europe BV, Zug, Switzerland). Experiments were performed between passage 4–6 as described earlier [20].

All subsequently described experiments were performed in quadruplicate.

### 2.2. Cell Treatment

Confluent cell layers were deprived of serum for 24 h for cell cycle synchronization and quiescence. Thereafter, the cells were incubated with increasing concentration (1–100 µg/mL) of either zinc iodide dissolved in dimethyl sulfoxide (ZnI-DMSO) or ZnI (cat. 223883, Merck & Cie, Buchs, Switzerland) or DMSO (cat. 223883, Merck & Cie) for 24 or 48 h. The final concentration of DMSO was kept constant in all experiments at 0.1%.

### 2.3. MMP-ELISA

Secreted MMPs were detected by commercially available ELISA kits (MMP-2: ab100606; MMP-9: ab246539; both purchased from Abcam, Cambridge, UK) in samples of cell culture medium collected as described below. ELISAs were performed as advised by the distributor.

### 2.4. Zymography

MMPs were determined in cell culture medium collected from 96-well plates before cells were fixed for collagen and fibronectin measurements described below. Medium was frozen immediately at −20 °C for later analysis. MMP-2 and MMP-9 activity was performed by gelatin zymography as described by Wilkesman and Kurz [21].

### 2.5. Collagen Type I and Fibronectin Deposition by Cell-Based ELISA

Cells were seeded into a 96-well plate and grown to confluence, before being serum-deprived for 24 h. Cells were stimulated with TGF-β1 (5 ng/mL) for 30 min, before either ZnI-DMSO, or ZnI, or DMSO was added at increasing concentrations. Cells were further incubated under standard conditions for 24 and 48 h. The cells were washed 3× with PBS and fixed for 2 × 5 min in 4% formaldehyde and washed once more with PBS. Unspecific antibody binding was reduced by incubating the fixed cells for 1 h with PBS containing 0.01% TWEEN-20 and 2% bovine serum albumin (blocking buffer). Collagen type I or fibronectin was detected by incubating the cells in a blocking buffer containing an antibody specific to either collagen type I (ab34710, Abcam, diluted 1:5000) or fibronectin (ab23750, Abcam, diluted 1:5000) overnight at 4 °C. After 3 washes with blocking buffer, the cells were incubated for 1 h with the species-specific secondary horseradish oxidase-labeled antibody (cat. 7076, Cell Signaling Technology, Danvers, MA, USA). Following 3 washes with blocking buffer and 1 wash with PBS, the substrate TMB (#T0440, Sigma-Aldrich, Buchs, Switzerland) was added and incubated at room temperature for 20 min. The color reaction was stopped by adding 50 µL of 0.1 M HCl, and the absorption was read in an ELISA plate reader at 450 nm as described earlier [22].

### 2.6. Soluble Collagen

Soluble collagen was detected in cell culture medium using a commercially available hydroxyproline assay kit (cat MAK008-1KT, Merck & Cie). Cell culture medium was collected from 96-well plates before cells were fixed for collagen and fibronectin measurements described above. The medium was frozen immediately at −20 °C for later analysis. In some experiments, cells were treated with human recombinant 5 ng/mL TGF-β1 (5 ng/mL; cat. 240-B-010/CF, R&D Systems, Minneapolis, MN, USA). MMP-2 activity was inhibited by addition of a commercial available inhibitor for MMP-2/MMP-9 at 100–400 nM (cat 444241, Merck).

### 2.7. Western Blotting

Protein analysis of soluble MMP-2, collagen type I, and the housekeeping protein GAPDH (glyceraldehyde-3-phosphate dehydrogenase) was as described earlier in detail [19,20]. Proteins were diluted (1:1) with RIPA buffer (radio-immuno-precipitation assay buffer, BioRad, Basel, Switzerland). The protein concentration was determined by Bradford assay (BioRad), and equal-sized protein amounts were fractionated in a gradient PAGE gel (BioRad) for 1 h at 100 V before the proteins were transferred to PVDF membranes (BioRad). Relative protein levels were determined after overnight incubation with specific primary antibodies; the same antibodies described above for cell-based ELISA were used. Membranes were 3× washed with phosphate-buffered saline and then incubated with species-specific horseradish-labeled secondary antibodies (Abcam) for 1 h, before protein bands were visualized by means of an ECL substrate (Sigma-Aldrich, Basel, Switzerland).

### 2.8. Statistics

All experiments were performed independently on four different days, and each data point was determined in duplicate for each experiment. The null hypothesis was that neither ZnI alone nor ZnI-DMSO, nor DMSO, modified the deposition of collagen type I or fibronectin. Furthermore, it was assumed that any of the above supplementations modified the expression or activity of MMP-2 or MMP-9.

The null hypothesis was tested by application of Student’s *t*-test (2-tail, paired) and ANOVA, as stated in the figure legends. *p*-values < 0.5 were considered as significant.

## 3. Results

### 3.1. TGF-β1 Deposition of Collagen Type I, but Not Fibronectin, Is Reduced by Zinc Supplementation

**Figure 1:**
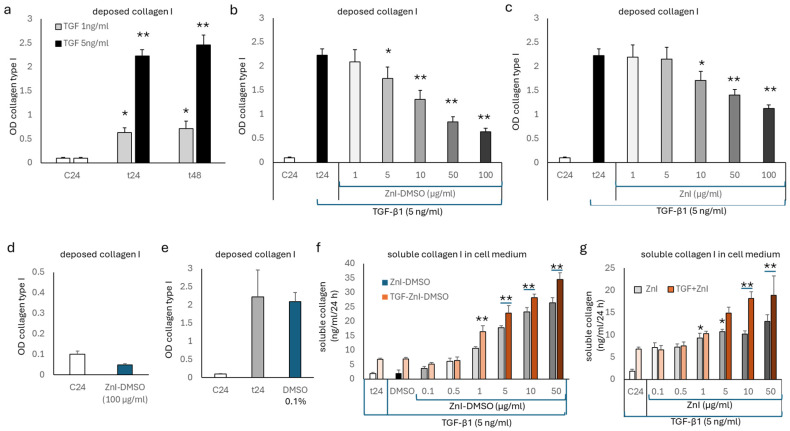
(**a**) Dose- and time-dependent stimulation of collagen type I deposition by human lung fibroblasts by TGF-β1 (5 ng/mL). (**b**) Concentration-dependent inhibition of TGF-β1 (5 ng/mL)-stimulated collagen type I deposition by ZnI-DMSO at 24 h. (**c**) Inhibitory effect of ZnI alone on TGF-β1 (5 ng/mL)-stimulated collagen type I deposition over 24 h. (**d**) Effect of ZnI-DMSO on spontaneous collagen type I deposition over 24 h. (**e**) Effect of DMSO alone on TGF-β1 (5 ng/mL)-stimulated collagen type I deposition over 24 h. (**f**) Effect of ZnI-DMSO on soluble total collagen content in cell culture medium. Darker color shades indicate increasing concentration. (**g**) Effect of ZnI alone on soluble total collagen content in cell culture medium. Darker color shades indicate increasing concentration. All experiments were performed in quadruplicate on different days; bar represents S.E.M. of all data. Statistics were calculated using Student’s *t*-test and ANOVA: * indicates *p*-value < 0.05, ** indicates *p*-value < 0.01. A line below an asterisk indicates the same *p*-value for both bars. C24: untreated cells; t24: TGF-β1-treated cells.

The TGF-β1 (5 ng/mL)-stimulated deposition of collagen type I was determined after 24 and 48 h (Figure 1a) by cell-based ELISA [19]. ZnI-DMSO reduced the TGF-β1-stimulated deposition of collagen type I in a concentration-dependent manner (1–100 µg/mL) at 24 h (Figure 1b) and at 48 h (Supplementary Figure S1). Similarly, ZnI alone reduced collagen type I deposition in a concentration-dependent manner but with a lower efficacy than ZnI-DMSO at 24 h (Figure 1c). ZnI-DMSO at the highest concentration (100 µg/mL) slightly reduced baseline deposition of collagen type I in untreated cells (Figure 1d). The solvent DMSO alone did not affect the TGF-β1-stimulated deposition of collagen type I (Figure 1e).

In parallel to the reducing effect of deposed collagen type I, ZnI-DMSO significantly increased the content of total collagen in the cell culture medium at 24 h (Figure 1f) and at 48 h (Supplementary Figure S2). Following a similar pattern, ZnI alone significantly increased the content of soluble total collagen in the cell culture medium at 24 h (Figure 1g). A similar effect was detected after 48 h (Supplementary Figure S1).

**Figure 2:**
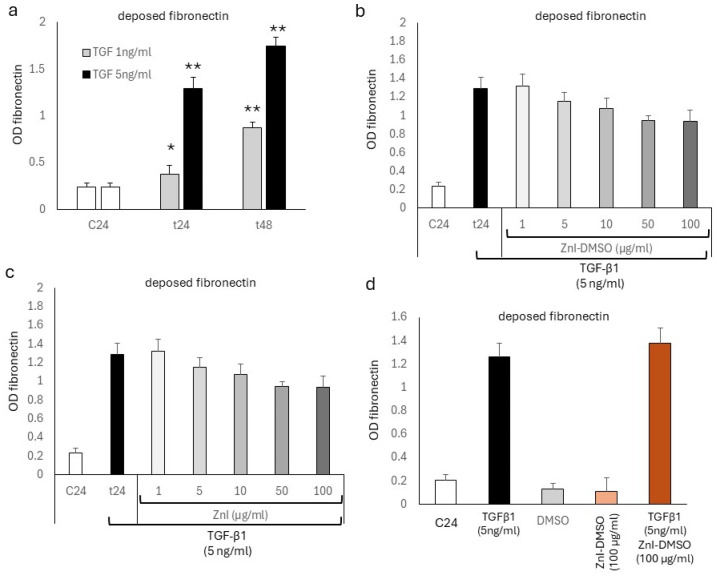
(**a**) Time- and concentration-dependent stimulation of fibronectin deposition by TGF-β1. (**b**) Effect of ZnI-DMSO on TGF-β1-stimulated fibronectin deposition at 24 h. (**c**) Effect of ZnI on TGF-β1-stimulated fibronectin deposition at 24 h. (**d**) Control conditions: effect of highest concentrations of either DMSO or ZnI-DMSO fibronectin deposition at 24 h. All experiments were performed in quadruplicate on different days; bar represents S.E.M. of all data. Statistics were calculated using Student’s *t*-test and ANOVA: * indicates *p*-value < 0.05, ** indicates *p*-value < 0.01. C24: untreated cells; t24: TGF-β1-treated cells.

Fibronectin deposition was stimulated by TGF-β1 (5 ng/mL) in a concentration-dependent manner at 24 h (Figure 2a) and 48 h (Supplementary Figure S3) and determined by cell-based ELISA. TGF-β1-induced deposition of fibronectin was only insignificantly reduced by ZnI-DMSO at 24 h (Figure 2b). Similarly, ZnI alone had no significant reducing effect on TGF-β1-induced fibronectin deposition (Figure 2c). In control experiments, neither DMSO nor ZnI, nor ZnI-DMSO, showed significant effects on spontaneous or TGF-β1 (5 ng/mL)-stimulated fibronectin deposition at 24 h.

### 3.2. Gelatinase Activity Is Up-Regulated by Zinc Supplementation and Regulates the Formation of Soluble Collagen

**Figure 3:**
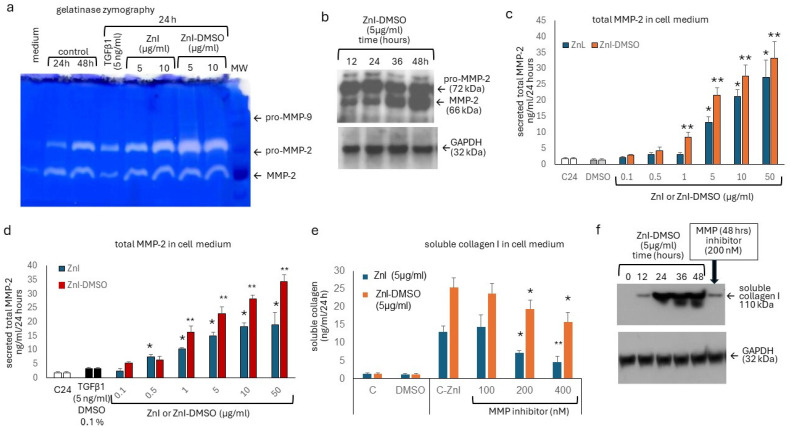
(**a**) Representative gelatin zymogram of cell culture medium collected at 24 h. Similar results were obtained in three additional experiments. (**b**) Representative Western blot showing the stimulatory effect of ZnI-DMSO on secreted pro-MMP-2 and active MMP-2 in cell culture medium over 48 h. GAPDH was used as housekeeping protein. (**c**) Effect of ZnI and ZnI-DMSO on secreted total MMP-2. (**d**) Effect of ZnI or ZnI-DMSO on secreted total MMP-2 in TGF-β1-stimulated cells. (**e**) MMP inhibition reduced the ZnI- and ZnI-DMSO-dependent increase of soluble total collagen in cell culture medium. (**f**) Representative Western blot of degraded soluble collagen type I as induced by ZnI-DMSO over 48 h, as well as the inhibitory effect on this process by a universal inhibitor of MMPs at 48 h. All experiments were performed in quadruplicate on different days; bar represents S.E.M. of all data. Statistics were calculated using Student’s *t*-test and ANOVA: * indicates *p*-value < 0.05, ** indicates *p*-value < 0.01. MW: molecular weight marker.

Next, we assessed the effect of ZnI-DMSO on MMP-2 and MMP-9 expression and activity. As shown in Figure 3a, MMP-2 expression increased with time in non-treated fibroblasts, while MMP-9 expression was minimal. Treatment with TGF-β1 reduced the activation of MMP-2 at 24 h, and both ZnI and ZnI-DMSO increased the expression and activity of MMP-2 in a concentration-dependent manner (Figure 3a).

The stimulatory effect of ZnI-DMSO on MMP-2 secretion and activation over 48 h was shown by Western blotting (Figure 3b). Furthermore, the increased secretion of total MMP-2 by ZnI-DMSO was confirmed by ELISA. Total MMP-2 secretion was concentration-dependently up-regulated by ZnI, and this was further increased when mixed with DMSO, which alone had no stimulatory effect (Figure 3c). The addition of DMSO significantly supported the stimulating effect of ZnI on MMP-2 expression at concentrations >1 µg/mL (Figure 3c), and a similar response of the cells was determined at 48 h (Supplementary Figure S4). A slightly reduced stimulatory effect of ZnI and ZnI-DMSO was determined in cells exposed to TGF-β1 (Figure 3d).

In an independent set of quadruplicate experiments, the effect of MMP-inhibition on the content of soluble collagen was assessed. As shown in Figure 3e, inhibiting MMPs significantly reduced the content of soluble collagen in the cell culture medium in a concentration-dependent manner for both ZnI (5 µg/mL) alone and ZnI-DMSO (5 µg/mL). Furthermore, the degradation of collagen type I and its increasing level in the cell culture medium in cells treated with ZnI-DMSO were confirmed by Western blotting (Figure 3f). In the presence of the universal MMP-inhibitor, this degradative effect of ZnI-DMSO was blocked (Figure 3f).

## 4. Discussion

This study supports the assumption that zinc supplementation has an anti-fibrotic or anti-remodeling effect on connective tissues such as peripheral lung fibroblasts. The data indicate that zinc supplementation reduces pro-inflammatory collagen type I through activating existing gelatinases.

Reviewed by Cheng and Chen, zinc deficiency is causatively involved in fibrotic disorders of most organs, and zinc supplementation was suggested as an add-on therapeutic strategy [23]. Zinc deficiency in mice lungs was associated with increased synthesis of collagen type I, fibronectin, and TIMPs, while that of MMPs was reduced [18]. However, the expression of MMPs and TIMPs was only determined on the mRNA level and not on enzyme activity. In another mouse model, cigarette smoke-induced inflammation and tissue remodeling were linked to the occurrence of zinc deficiency, which implies a role of zinc homeostasis in chronic obstructive pulmonary disease (COPD) [24]. In children with asthma low serum zinc levels were described, and it was assumed that this contributes to inflammation [25]. A similar correlation was observed in adult asthma patients [26]. In lung fibrotic diseases, low serum zinc levels were related to increased infection rate and reduced overall growth, but not to tissue remodeling [16,17]. The problem of linking zinc deficiency to tissue remodeling in chronic airway diseases lies in the fact that zinc is essential for the enzymatic function of many proteins [27,28].

In regard to extracellular matrix turnover and tissue remodeling, zinc has been shown to reduce the expression of pro-inflammatory collagen type I in mouse lungs [18]. A similar correlation between zinc deficiency and increased collagen synthesis was reported in a mouse model of renal fibrosis [29] as well as in myocardium fibrosis [30]. Reduced collagen expression after zinc supplementation was reported in several animal models of other fibrotic diseases but not in the lung [31,32]. Interestingly, zinc supplementation in patients with hepatic fibrosis increased the serum level of both MMP-2 and MMP-9, while that of collagen type II and type IV was decreased [33]. There is no study showing the direct the role of zinc-regulated gelatinase activity and reduced collagen deposition in fibrotic diseases. Thus, this study is the first to suggest such a direct effect of zinc supplementation on the activity of gelatinases as the cause of collagen degradation.

In our study, zinc supplementation had no significant effect on fibronectin deposition. In the context of chronic inflammatory lung diseases, fibronectin is essential for epithelial cell regeneration and wound repair [34,35,36]. Thus, the above increased deposition of fibronectin by TGF-β1, together with the degradation of collagen type I, might improve epithelial cell generation and wound repair in chronic inflammatory lung diseases. Other studies suggested that fibronectin is one of the factors that drives epithelial-to-mesenchymal transition, which would be pro-inflammatory [37]. Thus, the role of fibronectin in fibrotic diseases has to be studied in models that investigate the effect of zinc supplementation on the interaction of different cell types of the lung, such as epithelial cells and fibroblasts or immune cells.

This finding might be related to the role of zinc for wound healing and tissue repair in chronic inflammatory lung disease [38]. Zinc-DMSO mixtures had been suggested as an add-on therapeutic strategy for patients infected with SARS-CoV-2 [39]. The concept was tested in a randomized open trial including 200 SARS-CoV-2 patients. The patients treated with additional Afree (a locally registered inhalable medical drug) showed a significantly shorter time free of symptoms [40]. In a second study, the same researchers confirmed the beneficial effect of the compound [41].

The limits of this study are as follows: (i) The experiments were only performed in one human lung fibroblast line, and thus similar studies must be repeated in additional cell lines and in cells obtained from patients with different chronic inflammatory lung diseases, such as asthma, chronic obstructive pulmonary disease, and various types of lung fibrotic diseases. (ii) The effect of zinc supplementation in the context of lung fibrotic pathologies has to be investigated in future in situ studies in humans, since none of the available animal models of asthma, chronic obstructive pulmonary disease, and fibrosis reflects the full spectrum of the human disease [42,43,44]. However, studies in human patients might be limited by ethical aspects related to tissue sampling in diseases that do not require pathological tissue analyses, such as asthma or chronic obstructive pulmonary disease. (iii) The long-term effect of zinc supplementation on lung remodeling should be assessed.

However, the effect of zinc supplementation in wound repair might depend on the organ and the timing. Furthermore, the method by which zinc supplementation is performed might affect the outcome [45]. Considering the plethora of proteins and enzymes that are regulated by zinc ions or are involved in cross-cell membrane transport, it would be worthwhile to assess its effects on the level of transcriptomics, proteomics, and enzyme activity [46,47,48]. To understand the effect of zinc supplementation in chronic inflammatory lung diseases, new studies with a systematic approach are needed.

## 5. Conclusions

These findings imply new therapeutic strategies to prevent or reverse fibrotic lung diseases by means of zinc supplementation or gelatinase activation in the lung. It might be of interest to evaluate the effect of inhaled zinc salts together with cell-penetrating substances such as DMSO in chronic lung diseases.

## Data Availability

The raw data supporting the conclusions of this article will be made available by the authors on request.

## Supplementary Materials

The following supporting information can be downloaded at: https://www.mdpi.com/article/10.3390/biomedicines12061257/s1, Figure S1: Collagen type I deposition by ZnI-DMSO at 48 h; Figure S2: Collagen type I deposition by ZnI-DMSO at 48 h; content of soluble total collagen in cell culture medium 48 h after exposure to ZnI-DMSO and TGF-β1 stimulation; Figure S3: fibronectin deposition by ZnI-DMSO at 48 h; Figure S4: cell medium content of total MMP-2 at 48 h.

## Abbreviations

DMSO	dimethyl sulfoxide
ECM	extracellular matrix
ELISA	enzyme-linked immuno-sorbent assay
GAPDH	glyceraldehyde-3-phosphate dehydrogenase
MMP	matrix metalloproteinase
TGF-β1	transforming growth factor β1/tumor growth factor β1
ZnI	zinc iodide
