# Supplementary material for: Zinc Iodide Dimethyl Sulfoxide Reduces Collagen Deposition by Increased Matrix Metalloproteinase-2 Expression and Activity in Lung Fibroblasts
Source: Biomedicines. 2024 Jun 5;12(6):1257. doi: 10.3390/biomedicines12061257 (PMC11200730; doi:10.3390/biomedicines12061257)
Supplement: Supplementary file 1 [file biomedicines-12-01257-s001.zip › biomedicines-2991787-supplementary.pdf]

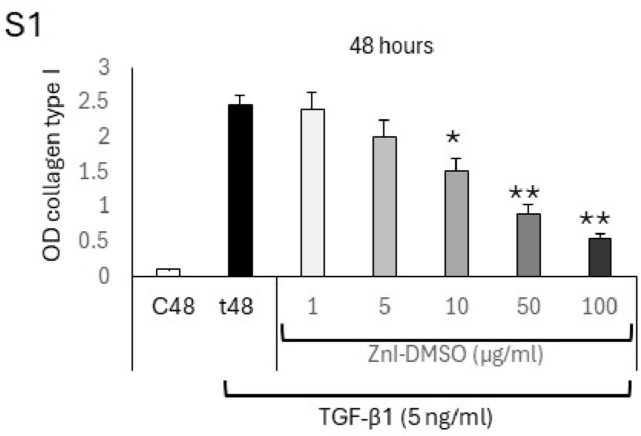

Figure S1: Collagen type I deposition by ZnI-DMSO at 48 h.

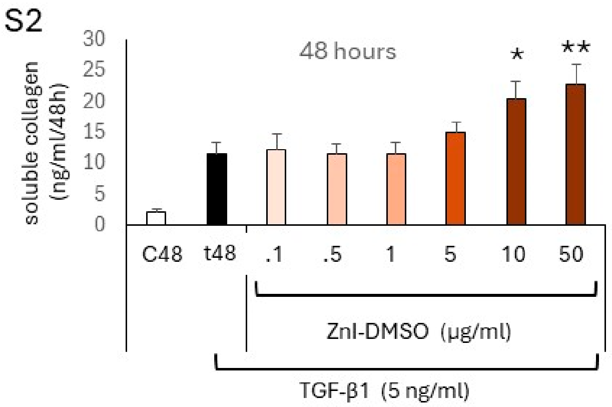

Figure S2: Collagen type I deposition by ZnI-DMSO at 48 h; content of soluble total collagen in cell culture medium 48 h after exposure to ZnI-DMSO and TGF-β1 stimulation.

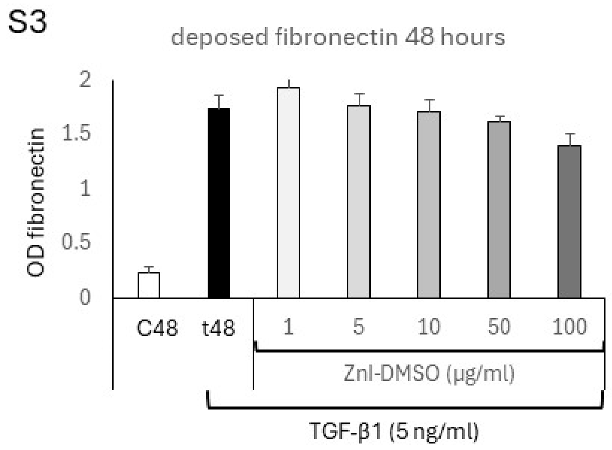

Figure S3: fibronectin deposition by ZnI-DMSO at 48 h.

S4

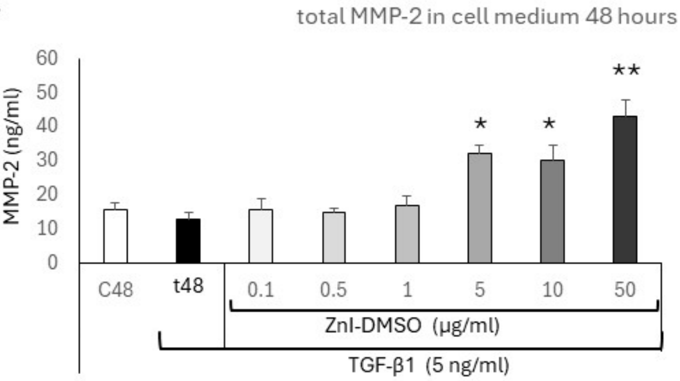

Figure S4: cell medium content of total MMP-2 at 48 h.
